# Supplementary material for: Pharmacological Modulation of Energy and Metabolic Pathways Protects Hearing in the Fus1/Tusc2 Knockout Model of Mitochondrial Dysfunction and Oxidative Stress
Source: Antioxidants (Basel). 2023 Jun 6;12(6):1225. doi: 10.3390/antiox12061225 (PMC10294946; doi:10.3390/antiox12061225)
Supplement: Supplementary file 1 [file antioxidants-12-01225-s001.zip › antioxidants-2378684-supplementary.pdf]

Figure S1

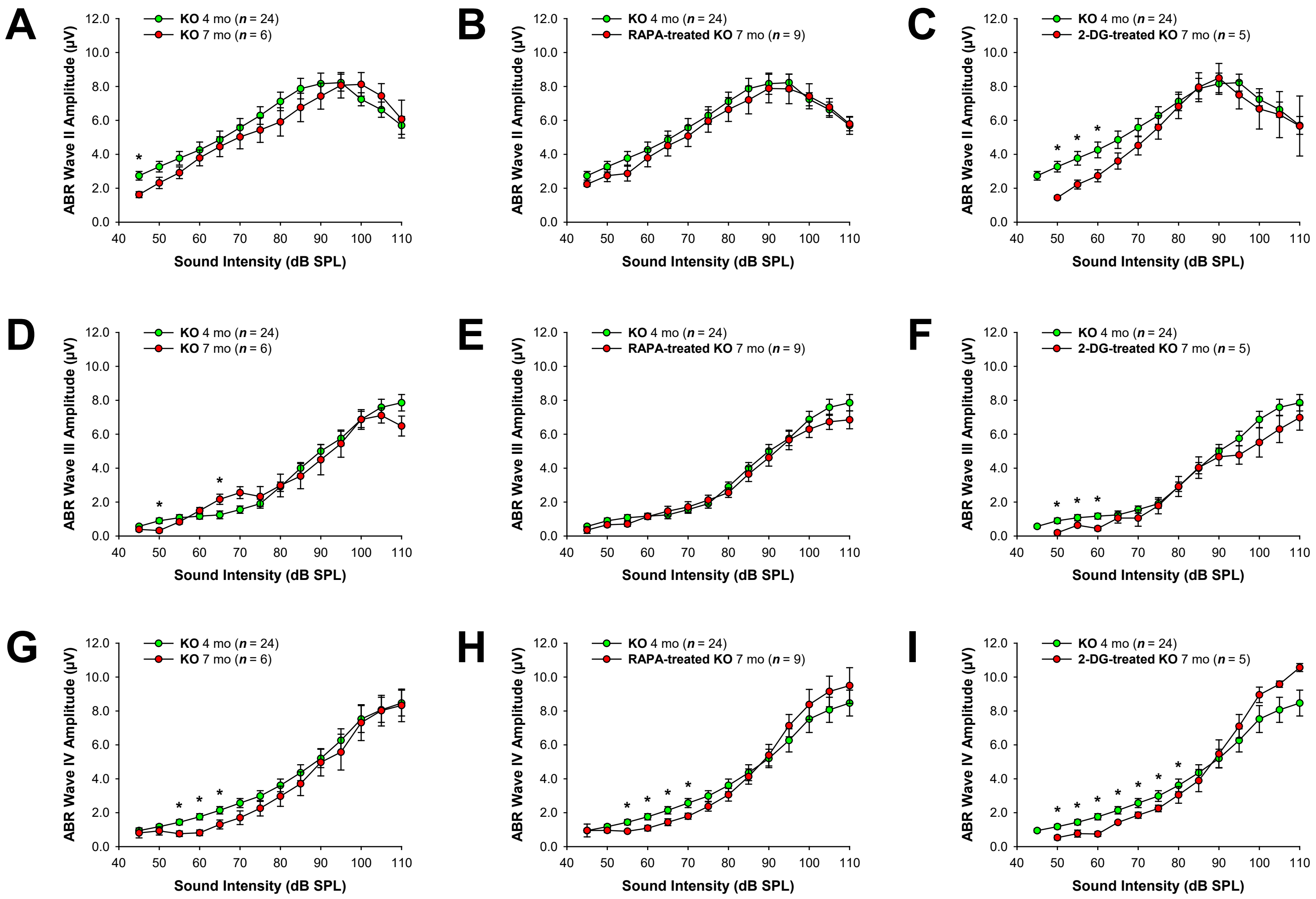

Figure S1: ABR wave II-IV amplitudes in rapamycin and 2-DG-treated Fus1 KO mice. (**A-C**) Shown are I/O function graphs of the average amplitude of ABR wave II as a function of sound intensity at 16 kHz in **A**) KO mice at 4 and 7 months of age; **B**) untreated and rapamycin-treated KO mice; **C**) untreated and 2-DG-treated KO mice. (**D-F**) Shown are I/O function graphs of the average amplitude of ABR wave III as a function of sound intensity at 16 kHz in **D**) KO mice at 4 and 7 months of age; **E**) untreated and rapamycin-treated KO mice; **F**) untreated and 2-DG-treated KO mice. (**G-I**) Shown are I/O function graphs of the average amplitude of ABR wave IV as a function of sound intensity at 16 kHz in **G**) KO mice at 4 and 7 months of age; **H**) untreated and rapamycin-treated KO mice; **I**) untreated and 2-DG-treated KO mice. Data are presented as mean ± SEM. *= $p < 0.05$, **= $p < 0.01$ (Student's t-test).

Figure S2

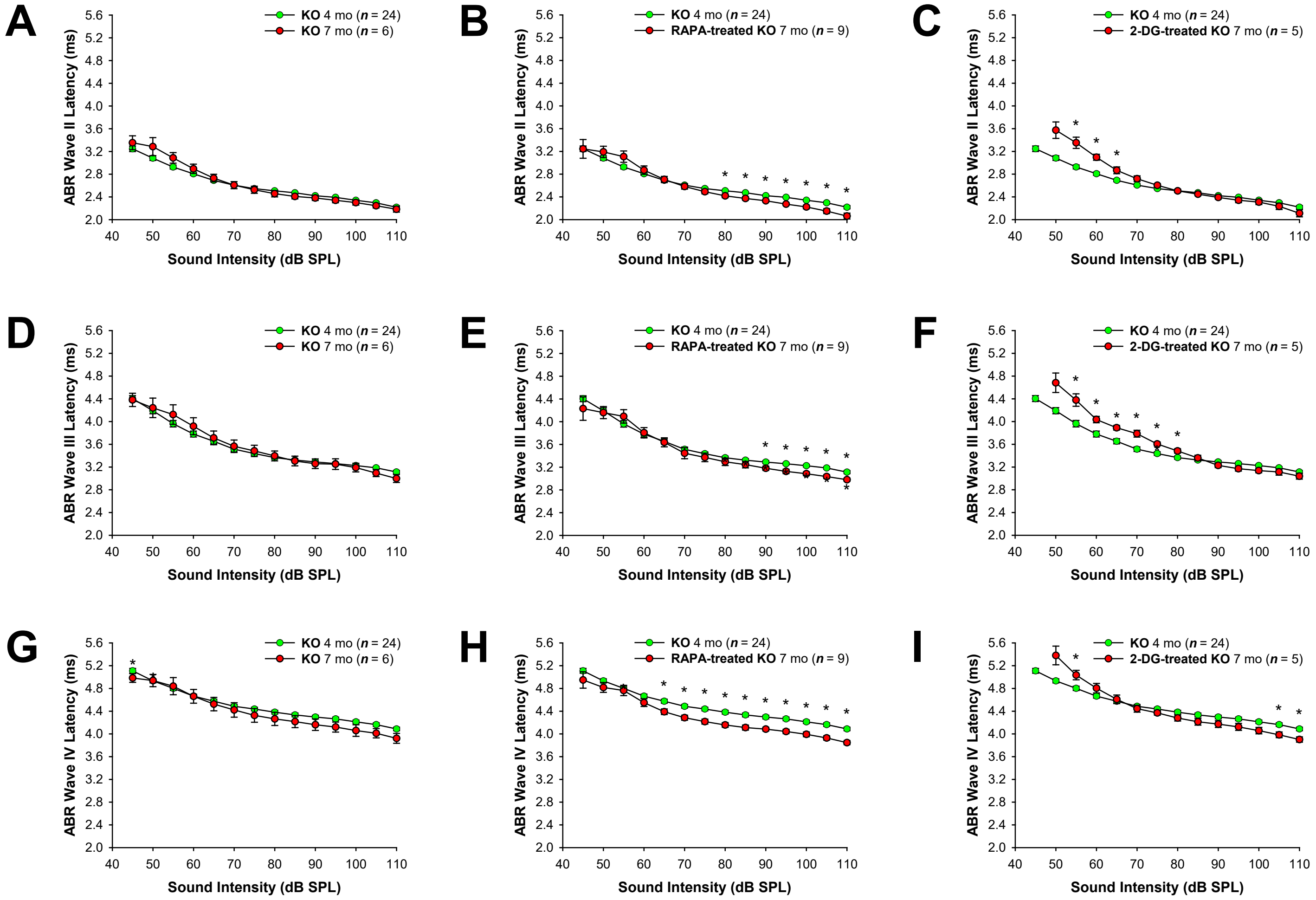

Figure S2: ABR wave II-IV latencies in rapamycin and 2-DG-treated Fus1 KO mice. (**A-C**) Shown are I/O function graphs of the average latency of ABR wave II as a function of sound intensity at 16 kHz in **A**) KO mice at 4 and 7 months of age; **B**) untreated and rapamycin-treated KO mice; **C**) untreated and 2-DG-treated KO mice. (**D-F**) Shown are I/O function graphs of the average latency of ABR wave III as a function of sound intensity at 16 kHz in **D**) KO mice at 4 and 7 months of age; **E**) untreated and rapamycin-treated KO mice; **F**) untreated and 2-DG-treated KO mice. (**G-I**) Shown are I/O function graphs of the average latency of ABR wave IV as a function of sound intensity at 16 kHz in **G**) KO mice at 4 and 7 months of age; **H**) untreated and rapamycin-treated KO mice; **I**) untreated and 2-DG-treated KO mice. Data are presented as mean ± SEM. *= $p < 0.05$, **= $p < 0.01$ (Student's t-test).
